# Thick *vs*. thin tongue coatings in hemodialysis patients: unveiling gut microbiome dysregulation and systemic health implications

**DOI:** 10.3389/fcimb.2025.1640429

**Published:** 2025-09-29

**Authors:** Yuqing Wang, Xueyan Zeng, Mengqi Wu, Bin Lu, Jiarui Wang, Saiping Chen, Aiping Zhang, Min Huang, Yanqin Zhu, Hong Liu, Fenggui Zhu, Shilei Chen, Xin Zhou, Luyang Zhao, Junyi Liu, Riyang Lin

**Affiliations:** ^1^ Department of Nephrology, Hangzhou Traditional Chinese Medicine (TCM) Hospital of Zhejiang Chinese Medical University, Hangzhou, Zhejiang, China; ^2^ Department of Nephrology, Sichuan Second Hospital of Traditional Chinese Medicine, Chengdu, Sichuan, China; ^3^ Xingqiao Street Community Health Service Center, Hangzhou, Zhejiang, China; ^4^ Department of General Medicine, Hangzhou Xihu District Zhuantang Street Community Health Service Centre, Hangzhou, Zhejiang, China; ^5^ Tianshui Wulin Street Community Heal Care Centre, Hangzhou, Zhejiang, China; ^6^ Donghu Street Community Health Service Center, Hangzhou, Zhejiang, China; ^7^ Key Laboratory of Kidney Disease Prevention and Control Technology, Hangzhou, Zhejiang, China

**Keywords:** hemodialysis, tongue coating, gut microbiota, oral-gut axis, gastrointestinal complications

## Abstract

**Background:**

Gastrointestinal (GI) disturbances are prevalent in maintenance hemodialysis (MHD) patients and are closely associated with gut microbiota dysregulation. Tongue coating thickness, a key diagnostic feature in traditional Chinese medicine, may reflect systemic and microbial health. This study aimed to explore the relationship between tongue coating phenotype and gut microbiota composition in MHD patients.

**Methods:**

A matched case-control study was conducted involving 30 MHD patients divided into thick (HTZ, n = 15) and thin (BTZ, n = 15) tongue coating groups, along with 15 healthy controls (DZZ). Fecal samples were analyzed via 16S rRNA sequencing to assess microbial diversity, taxonomic profiles, and predicted functional pathways.

**Results:**

Alpha-diversity indices were significantly lower in BTZ than in DZZ (q < 0.05), while no difference was found between HTZ and BTZ. Beta-diversity showed closer clustering between HTZ and BTZ than with DZZ. Compared to DZZ, both HTZ and BTZ exhibited reduced levels of genera typically associated with health or commensal functions (*Romboutsia, Subdoligranulum*) and increased abundances of taxa often linked to inflammation or disease (*Escherichia-Shigella, Ruminococcus gnavus*). Functional predictions indicated that HTZ was enriched in pathways related to disease processes and showed diminished cellular and metabolic functions.

**Conclusion:**

Tongue coating thickness in MHD patients reflects underlying gut microbial composition. Thick tongue coatings indicate a state of dysbiosis with potential health implications, whereas thin coatings are associated with a microbiota profile that may be more favorable. These findings support the potential use of tongue coating thickness as a noninvasive biomarker for gut health assessment in clinical nephrology.

## 1 Introduction

Hemodialysis is a life-sustaining treatment for patients with end-stage renal disease (ESRD), yet it is often accompanied by a range of complications that significantly impact patients’ quality of life and prognosis. Gastrointestinal (GI) problems have emerged as a prevalent and complex issue. Studies have consistently shown that hemodialysis patients frequently suffer from various GI symptoms. For instance, research by Doğu Karahan and İdris Şahin indicated that dyspepsia, nausea, and epigastric pain were highly common, affecting 50%, 45%, and 44% of the patients in their study cohort respectively (Karahan and Şahin, 2022). Additionally, Sang Cheol Park et al. found that constipation was present in 25.9% of hemodialysis patients and was associated with an increased risk of cardiovascular events and all-cause mortality (Park et al., 2025). These GI manifestations not only disrupt patients’ daily lives but also pose challenges to the effectiveness of hemodialysis treatment. A recent systematic review involving over 5,000 dialysis patients confirmed that constipation, indigestion, abdominal pain, and reflux are among the most prevalent GI symptoms in this population, and highlighted their adverse effects on quality of life and clinical outcomes (Zuvela et al., 2018).

Hemodialysis patients exhibit significant gut microbiota dysregulation. Key findings include reduced α-diversity (e.g., Shannon and Chao1 indices) and altered microbial composition, with depletion of beneficial bacteria such as short-chain fatty acid (SCFA)-producing *Faecalibacterium*, *Bifidobacterium*, and *Akkermansia muciniphila*, and overgrowth of pathobionts like *Enterobacteriaceae* and *Klebsiella* (Wang et al., 2025). Metabolic dysfunction drives excessive production of uremic toxins (indoxyl sulfate, p-cresyl sulfate, trimethylamine N-oxide) from tryptophan and choline metabolism, while SCFA deficiency impairs intestinal barrier integrity and triggers systemic inflammation via TLR4/NF-κB signaling (Regis et al., 2025). A recent review emphasized how these microbiota alterations contribute to systemic inflammation, cardiovascular risk, and malnutrition in ESRD patients, and noted that microbiome-targeted therapies (e.g., probiotics, prebiotics) may mitigate dialysis-related complications (Velasquez et al., 2018). Clinically, this dysregulation correlates with increased cardiovascular risk (vascular calcification, atherosclerosis), malnutrition, sarcopenia, and gut motility disorders (e.g., constipation), with severity linked to dialysis duration and toxin levels (Shao et al., 2025).

Our team’s previous research discovered significant differences in the oral microbiota between maintenance hemodialysis (MHD) patients with thick and thin tongue coatings (Zhu et al., 2025). The overall microbial species richness in MHD patients with thick tongue coatings is higher compared to those with thin tongue coatings, with distinct differences in microbiota abundance at various taxonomic levels. For example, the abundance of genera such as *Prevotella* and *Megasphaera* is significantly higher in patients with thick tongue coatings, suggesting that tongue coating thickness (TCT) may be related to the composition and diversity of the oral microbiome in MHD patients.

Multiple studies have highlighted the relationships between the tongue coating microbiota and gut microbiota. In healthy individuals, there is a positive correlation in the relative abundance of *Prevotella* in both the tongue coating and intestines (Guo et al., 2022). In disease states, such as IBS-D and MAFLD, changes in tongue coating microbiota are accompanied by alterations in gut microbiota (Lu et al., 2022; Tang et al., 2023). Systemic diseases like COVID-19 and schizophrenia also lead to concurrent changes in oral and gut microbiomes (Cui et al., 2022; Ling et al., 2023), and age-related increases in oral bacteria translocation to the gut have been observed (Iwauchi et al., 2019). A recent comprehensive review on the oral–gut axis described mechanisms including microbial migration, metabolite signaling, and immune modulation, and linked these findings to diseases such as IBD, colorectal cancer, and cardiometabolic disorders (Park et al., 2021). Another narrative review emphasized that oral pathogens (e.g., *Fusobacterium*, *Streptococcus*) may translocate and colonize the gut, contributing to gastrointestinal and systemic pathologies (Sulaiman et al., 2024). These findings imply that the oral-gut axis plays a crucial role in microbial interactions, with potential implications for disease mechanisms.

Given the established differences in oral microbiota between thick and thin tongue coatings in hemodialysis patients, and the reported connections between oral and gut microbiota, a critical question remains: do hemodialysis patients with thick and thin tongue coatings exhibit differences in gut microbiota composition and function? This study aims to investigate the structural and functional disparities in gut microbiota between these two groups, exploring whether tongue coating thickness, as a marker of oral microecology, is associated with gut microecological variations. This question is clinically relevant because recent intervention trials targeting gut microbiota (e.g., high-fiber diets, synbiotics) in ESRD patients have demonstrated reductions in uremic toxins and inflammation, suggesting potential synergy if oral markers could guide interventions (Kuskunov et al., 2023). By addressing this gap, we seek to deepen our understanding of the oral-gut axis in hemodialysis patients and provide a scientific basis for microecology-targeted interventions to improve GI outcomes in this population.

## 2 Methods

### 2.1 Study design and sample selection

This study employed a matched case-control design, including 30 maintenance hemodialysis (MHD) patients (15 with thick tongue coating [HTZ], 15 with thin tongue coating [BTZ]) and 15 healthy controls (DZZ). Stool samples were collected concurrently with tongue coating assessments between December 2022 and June 2023 at Hangzhou TCM Hospital of Zhejiang Chinese Medical University, following the same inclusion/exclusion criteria as the original study (Zhang et al., 2024), following the inclusion criteria of ≥ 3 months of stable MHD treatment, age > 18 years, and no antibiotic use within the past month. Exclusion criteria included organic gastrointestinal disorders, recent probiotic use, or acute infections. Specifically, HTZ was defined as a tongue coating thickness (TCT) score ≥ 24 (thick coating), BTZ as 5-13 (thin coating), and DZZ as healthy volunteers with TCT scores within the moderate range (14-23). These healthy volunteers were specifically selected to have tongue coating thickness within the moderate range, in order to represent typical healthy conditions and to avoid including extremely thin or thick coatings. We did not further stratify the healthy control group into thick or thin coating subgroups because such extreme tongue coatings are rarely observed in healthy populations. This approach helps to avoid potential interference from atypical conditions in the interpretation of results.

### 2.2 Tongue coating image acquisition and assessment

**Figure 1:**
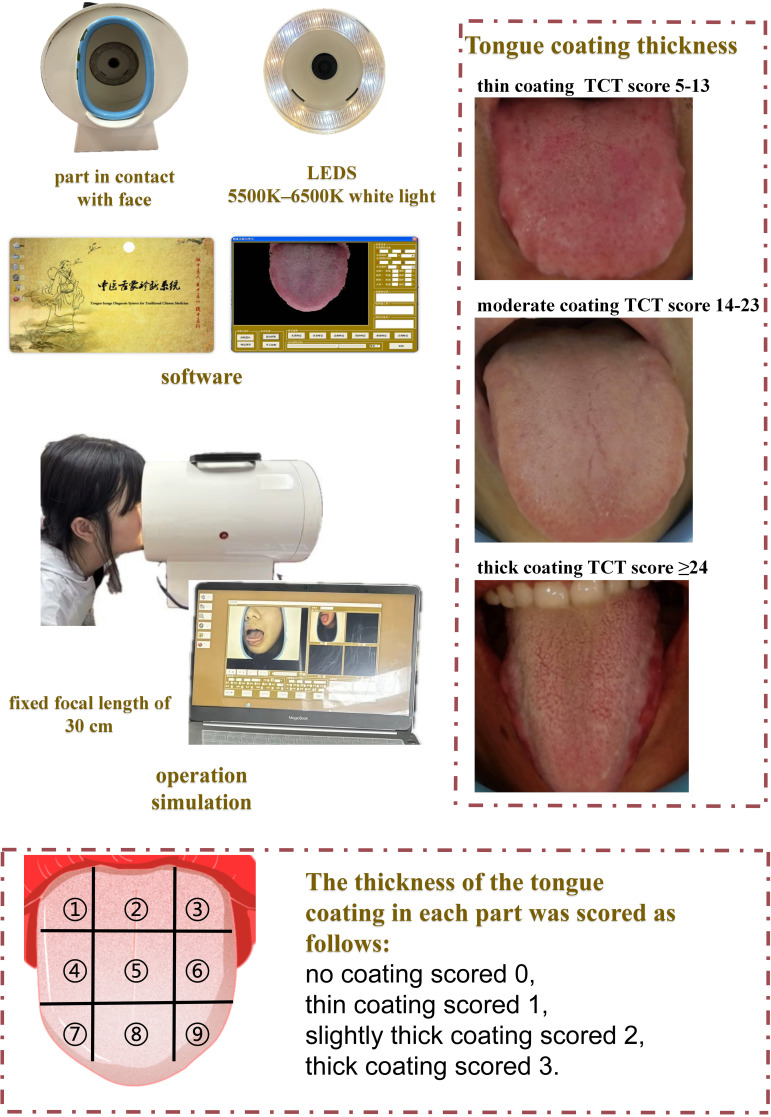
Components and operation simulation of a tongue diagnosis system.

Tongue images were obtained using the TCM Tongue Diagnostic Expert System with standardized lighting (5500–6500K white light) and a fixed focal length of 30 cm (**Figure 1**). All subjects were imaged in the morning before eating or oral hygiene to minimize dietary effects. Each participant sat upright and extended the tongue naturally (avoiding curling or strain) for the photograph. Two licensed TCM practitioners, blinded to each other’s ratings, independently assessed tongue coating thickness (TCT) on these images. Following Shimizu et al. (2007) (Shimizu et al., 2007) and the Japanese “Shimada” scheme, we divided the tongue dorsum into nine regions (three transverse zones: anterior, middle, posterior; each subdivided into left, center, and right). Each region was scored on an ordinal 0–3 scale (a modified version of Shimada’s criteria): 0 = no visible coating; 1 = thin coating (papillae clearly visible through the coating); 2 = moderate coating; 3 = thick coating (papillae not visible). The total TCT score for each subject was calculated as the sum of all nine regional scores (Zhu et al., 2025).

This scoring system has empirical support: Shimizu et al. demonstrated that their Tongue Coating Index (a similar 0–2 scale) correlates with tongue microbial burden. In other words, higher coating scores correspond to greater anaerobic bacterial counts on the tongue surface. To ensure reproducibility, we trained the practitioners in the above criteria before scoring and calculated inter-rater reliability by weighted Cohen’s kappa. In our study, the overall κ was 0.817 (regional κs ranged 0.631–0.876), indicating substantial-to-almost-perfect agreement. These values confirm that the nine-region TCT scoring is both scientifically grounded and consistent between observers.

### 2.3 Stool sample retrieval

Frozen stool samples stored at −80 °C since the original study (Zhang et al., 2024) will be utilized. Sample collection mirrored the parent study’s protocols: fasting participants provided stool samples in sterile tubes within 2 hours of collection, prior to dialysis or oral hygiene, to minimize contamination.

### 2.4 Microbiome analysis

#### 2.4.1 DNA extraction

Genomic DNA was extracted using the CTAB method (Simpson, 2010). Briefly, fecal samples were lysed with CTAB buffer and glass beads, followed by phenol-chloroform extraction (Sambrook and Russell, 2006) and ethanol precipitation. DNA purity (A260/A280 = 1.8–2.0) and concentration (> 10 ng/μL) were verified by agarose gel electrophoresis and Nanodrop (Glasel, 1995).

#### 2.4.2 16S rRNA sequencing

The V3-V4 hypervariable regions of the 16S rRNA gene were amplified using barcoded primers 338F and 806R. Each PCR reaction utilized 50 ng of template DNA under the following cycling conditions: initial denaturation at 95 °C for 3 min, 25 cycles of 95 °C for 30 s, 55 °C for 30 s, 72 °C for 45 s, and a final extension at 72 °C for 10 min. Amplified products were purified with AMPure XT beads and subsequently sequenced on an Illumina NovaSeq 6000 platform (2×250 bp paired-end configuration) by Hangzhou Lianchuan Biotechnology (Matsumoto and Sugano, 2013).

#### 2.4.3 Bioinformatics pipeline

Raw sequencing reads were processed in QIIME 2 (Bolyen et al., 2019) using DADA2 (Callahan et al., 2016) to remove low-quality sequences (Q < 20), denoise, and generate ASVs. For taxonomic annotation, ASVs were compared against the SILVA database (version 138) at 97% identity, with chimeric sequences removed. Due to limited resolution of species-level annotation based on the V3-V4 region of the 16S rRNA gene, species names used in this study represent putative assignments based on the best available database match and should be interpreted with caution.

In diversity analysis, α-diversity was assessed using Chao1, Observed ASVs, Shannon, and Simpson indices to evaluate richness and evenness, with group comparisons conducted via Wilcoxon tests. For beta diversity, PCoA and NMDS were performed based on Bray-Curtis and Unweighted Unifrac distances to visualize compositional differences, with statistical significance tested using PERMANOVA (999 permutations) (Kelly et al., 2015).

Differential abundance of taxa between groups was identified using LEfSe (Segata et al., 2011) with a logarithmic LDA score threshold > 2.0 and Wilcoxon tests, applying FDR correction for multiple comparisons. Functional prediction of KEGG pathways from ASV data was carried out using PICRUSt2 (Douglas et al., 2020), with STAMP (Parks and Beiko, 2010) software comparing pathway abundances (t-test, FDR < 0.05).

### 2.5 Statistical analysis

Clinical demographic variables (age, sex) and clinical parameters (Kt/V, hs-CRP) were compared using t-tests (parametric) or Wilcoxon tests (non-parametric). For microbiome analyses conducted in R (v4.3.0) with phyloseq (McMurdie and Holmes, 2013) and ggplot2 (Wen et al., 2023), species differences were assessed using Fisher’s exact test (no biological replicates), Mann-Whitney U test (two-group comparisons with replicates), or Kruskal-Wallis test (multi-group comparisons with replicates). Differential abundance tests report both p and FDR-adjusted q values; statistical significance was defined as q < 0.05. Nominal (unadjusted) p-values are reported only where noted.

### 2.6 Ethical considerations

The study was approved by the Research Ethics Committee of Hangzhou Hospital of TCM (approval number: 2023ZL110), with all participants providing written informed consent. Data were anonymized, and sample reanalysis followed the original study’s ethical guidelines.

## 3 Results

### 3.1 Species annotation analysis

**Figure 2:**
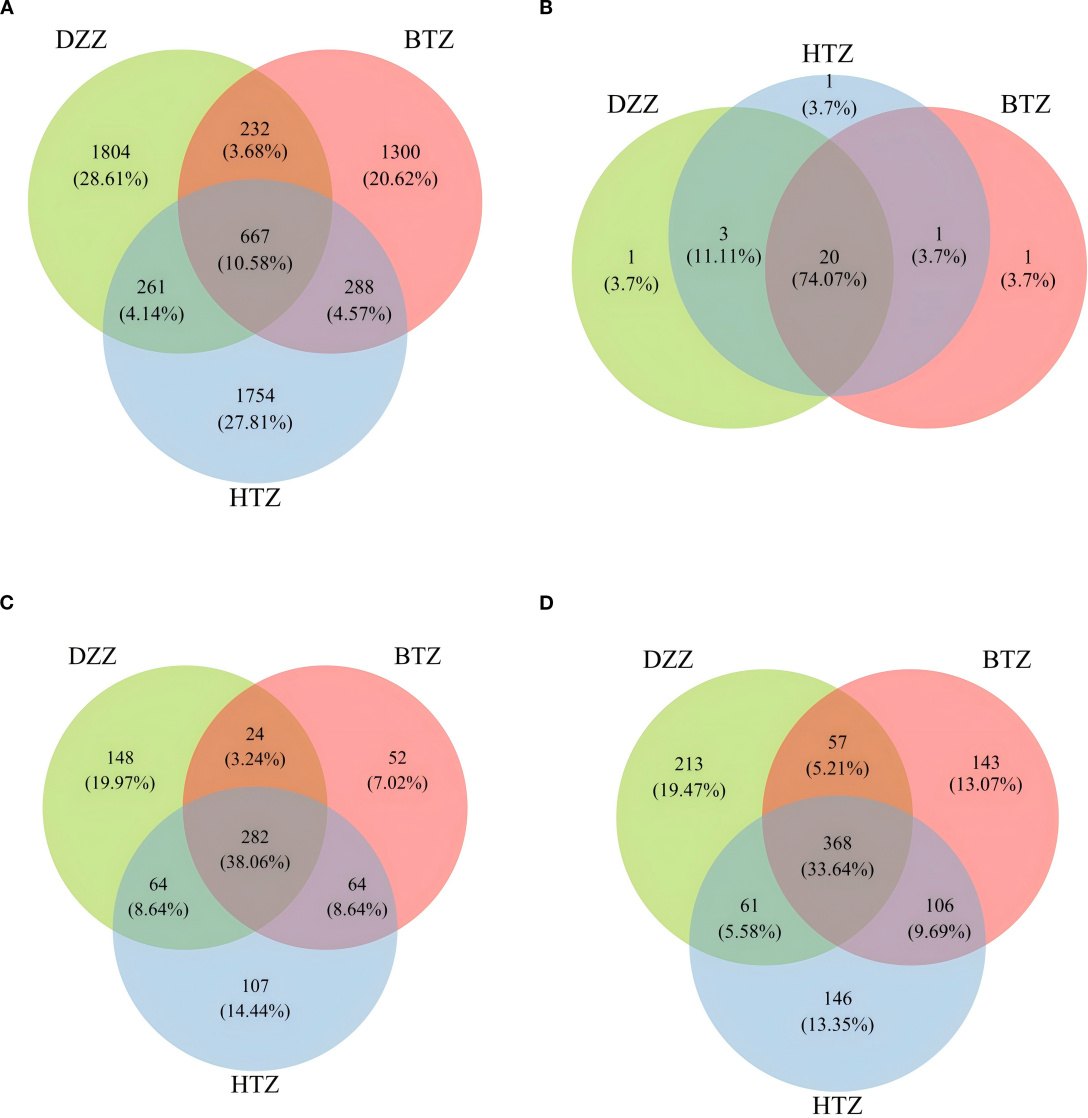
**(A)** Venn diagram of ASVs among DZZ, BTZ, and HTZ groups. A total of 6306 ASVs were detected. Among them, 667 (10.6%) were shared by all three groups, while DZZ, BTZ, and HTZ uniquely contributed 1804 (28.6%), 1300 (20.6%), and 1754 (27.8%) ASVs, respectively. Shared ASVs between two groups only accounted for a small proportion. **(B)** Venn diagram of bacterial phyla among DZZ, BTZ, and HTZ groups. A total of 23 phyla were detected. 20 (87.0%) were shared by all groups, and each group had one unique phylum (4.3%). No phylum was shared between only two groups, indicating high similarity at the phylum level. **(C)** Venn diagram of bacterial genera among DZZ, BTZ, and HTZ groups. A total of 589 genera were detected. 282 (47.9%) were shared by all groups, while DZZ, BTZ, and HTZ had 148 (25.1%), 52 (8.8%), and 107 (18.2%) unique genera, respectively. This reflects both shared and group-specific microbial features at the genus level. **(D)** Venn diagram of bacterial species among DZZ, BTZ, and HTZ groups. A total of 870 species were detected. 368 (42.3%) were shared by all groups, while DZZ, BTZ, and HTZ had 213 (24.5%), 143 (16.4%), and 146 (16.8%) unique species, respectively. This indicates high species-level diversity and group specificity.

According to the ASV abundance tables obtained from sequencing fecal samples of three groups, there were 2,970 ASVs in the HTZ group, 2,487 ASVs in the BTZ group, and 2,964 ASVs in the DZZ group. Notably, HTZ fecal samples contained the highest number of ASVs, while BTZ had the lowest, highlighting individual differences in the intestinal microbiota. The Venn diagram showed that HTZ and DZZ shared 928 ASVs, with HTZ having 2,042 unique ASVs and DZZ having 2,036 unique ASVs. BTZ and DZZ shared 899 ASVs, with BTZ having 1,588 unique ASVs and DZZ having 2,065 unique ASVs. HTZ and BTZ shared 955 ASVs, with HTZ having 2,015 unique ASVs and BTZ having 1,532 unique ASVs. All three groups (HTZ, BTZ, and DZZ) shared 667 ASVs. Preliminary results indicated significant differences in intestinal microbiota composition among the thick tongue coating group (HTZ), thin tongue coating group (BTZ), and healthy control group (DZZ) of hemodialysis patients. Specifically, the HTZ group had a greater variety of intestinal microbiota, while the BTZ group and healthy control group had relatively fewer. Notably, the intestinal microbiota of the HTZ group shared more common species with the BTZ group (**Figure 2**).

### 3.2 Species diversity analysis

#### 3.2.1 α-diversity analysis

**Figure 3:**
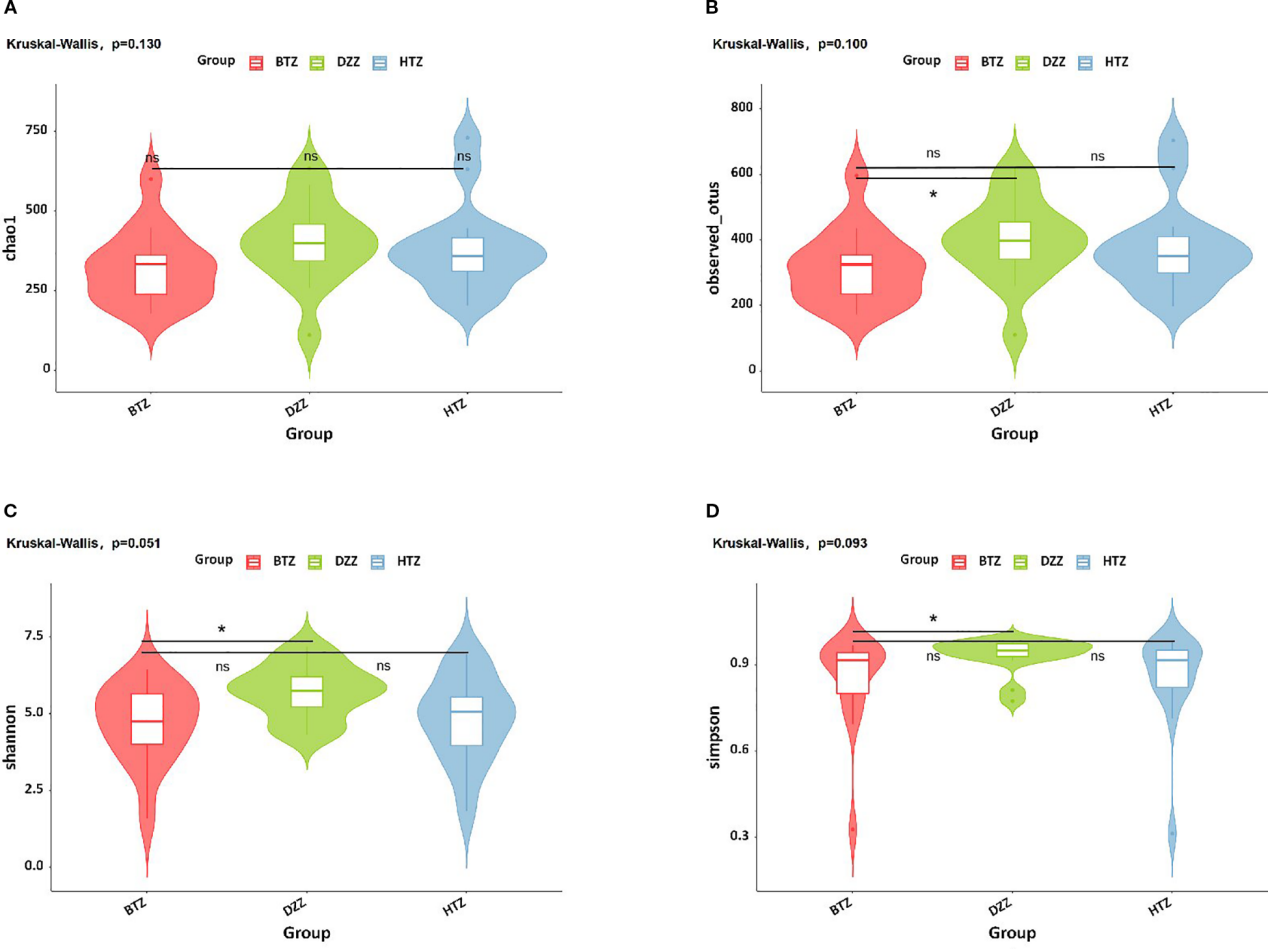
α-diversity. Violin plots show **(A)** Chao1, **(B)** Observed species, **(C)** Shannon, and **(D)** Simpson indices. No significant difference in Chao1 index was found among groups. DZZ had higher Observed species, Shannon, and Simpson indices than BTZ, with no difference between HTZ and BTZ. Healthy controls (DZZ) exhibited higher microbiota richness and evenness than the thin tongue coating group (BTZ) of hemodialysis patients. (ns = not significant; * indicates q < 0.05).

According to the results shown in the violin plot, there was no statistically significant difference in the Chao1 index among the three groups (p > 0.05). The Observed species (q = 0.04), Shannon (q = 0.03), and Simpson indices (q = 0.03) showed that the DZZ values were higher than those of BTZ, while no significant difference was observed between HTZ and BTZ (p > 0.05). This indicates that the intestinal microbiota richness and evenness of the healthy control group were higher than those of the thin tongue coating group of hemodialysis patients. The lack of significant differences between HTZ and DZZ in α-diversity may reflect preserved overall microbial diversity in some HD patients with thick tongue coating. However, as shown in subsequent β-diversity and taxonomic composition analyses, the microbiota community structure and dominant taxa differed between HTZ and DZZ, suggesting compositional alterations despite similar richness and evenness (**Figure 3**).

#### 3.2.2 β-diversity analysis

**Figure 4:**
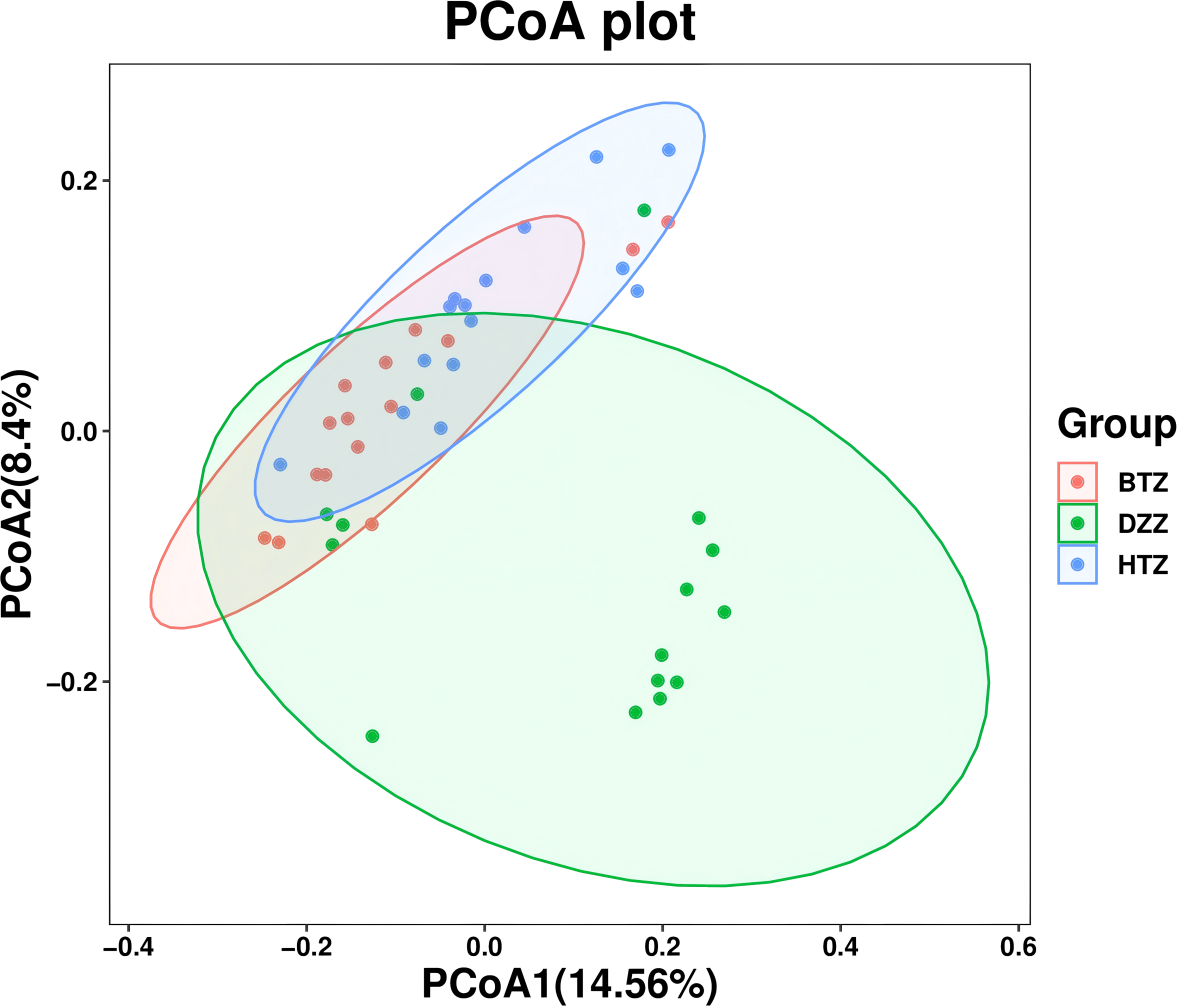
β-diversity. PCoA plot based on unweighted UniFrac distances. The PCoA plot illustrates the microbial community structure among HTZ, BTZ, and DZZ groups. HTZ and BTZ samples cluster closely, indicating similar microbiota composition, while both are clearly separated from the DZZ group (healthy controls), suggesting marked compositional differences (q = 0.01). (PCoA1 = 14.56%, PCoA2 = 8.4%).

PCoA based on unweighted UniFrac distances was used to visualize differences in microbial community composition among the three groups. The PCoA plot showed that the HTZ and BTZ groups clustered closely together, while both were clearly separated from the DZZ group, indicating distinct differences in intestinal microbiota composition between dialysis patients and healthy controls (PCoA1 = 14.56%, PCoA2 = 8.4%, q = 0.01). These results suggest that the overall microbial communities of HTZ and BTZ patients were more similar to each other and differed markedly from those of healthy individuals (**Figure 4**).

### 3.3 Species difference analysis

#### 3.3.1 Composition heatmap

**Figure 5:**
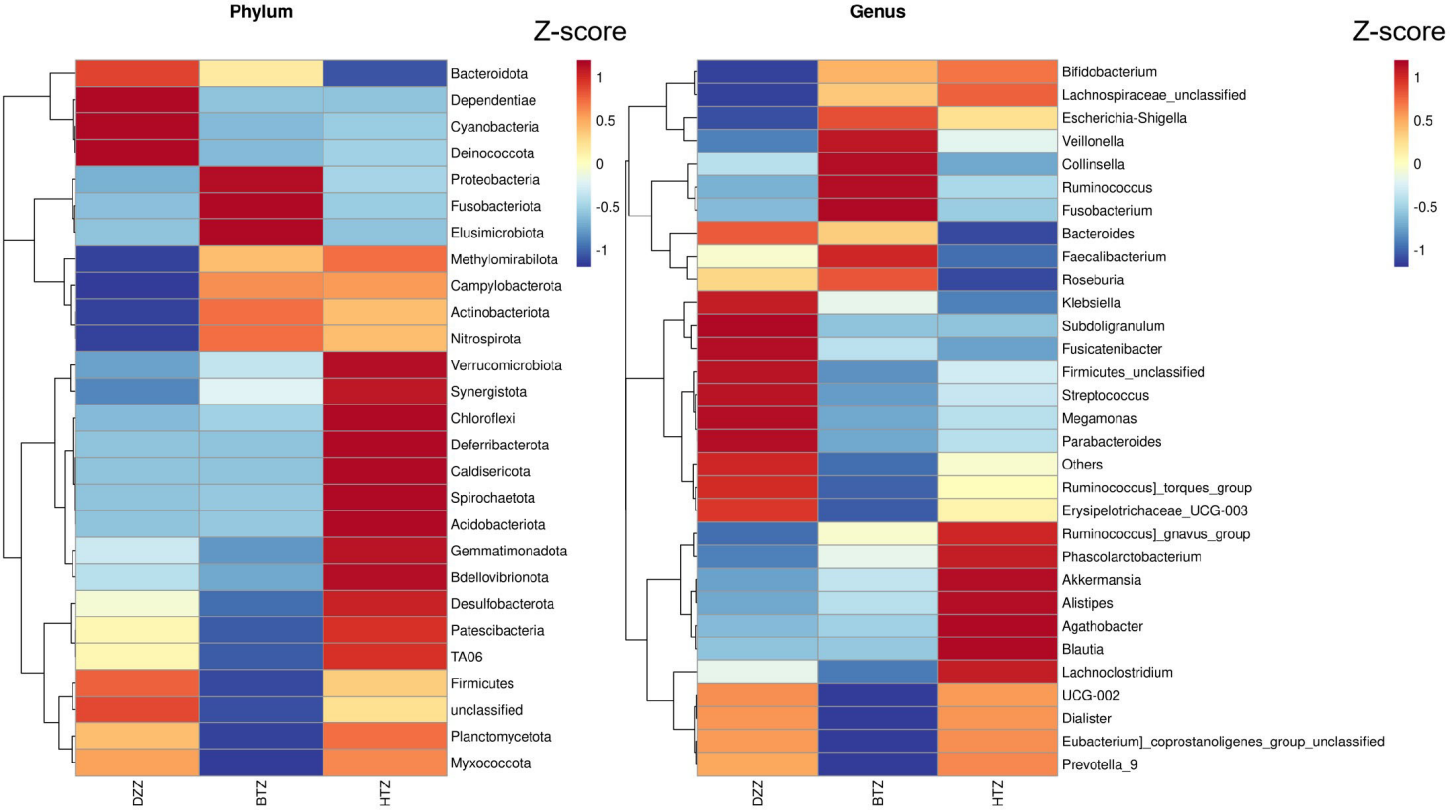
Phylum-level and genus-level composition heatmap. Heatmap of the top 30 most abundant bacterial phylum and genera across the HTZ, BTZ, and DZZ groups. Colors represent Z-score normalized relative abundances. Differences in phylum and genus distribution patterns indicate compositional divergence between groups.

Clustering heatmaps visually demonstrated the similarities and differences in microbiota composition among groups at various taxonomic levels. **Figure 5** shows significant differences in the composition and distribution of intestinal microbiota at both the genus and phylum levels among the HTZ, BTZ, and DZZ groups.

#### 3.3.2 Comparison of taxonomic abundance

**Figure 6:**
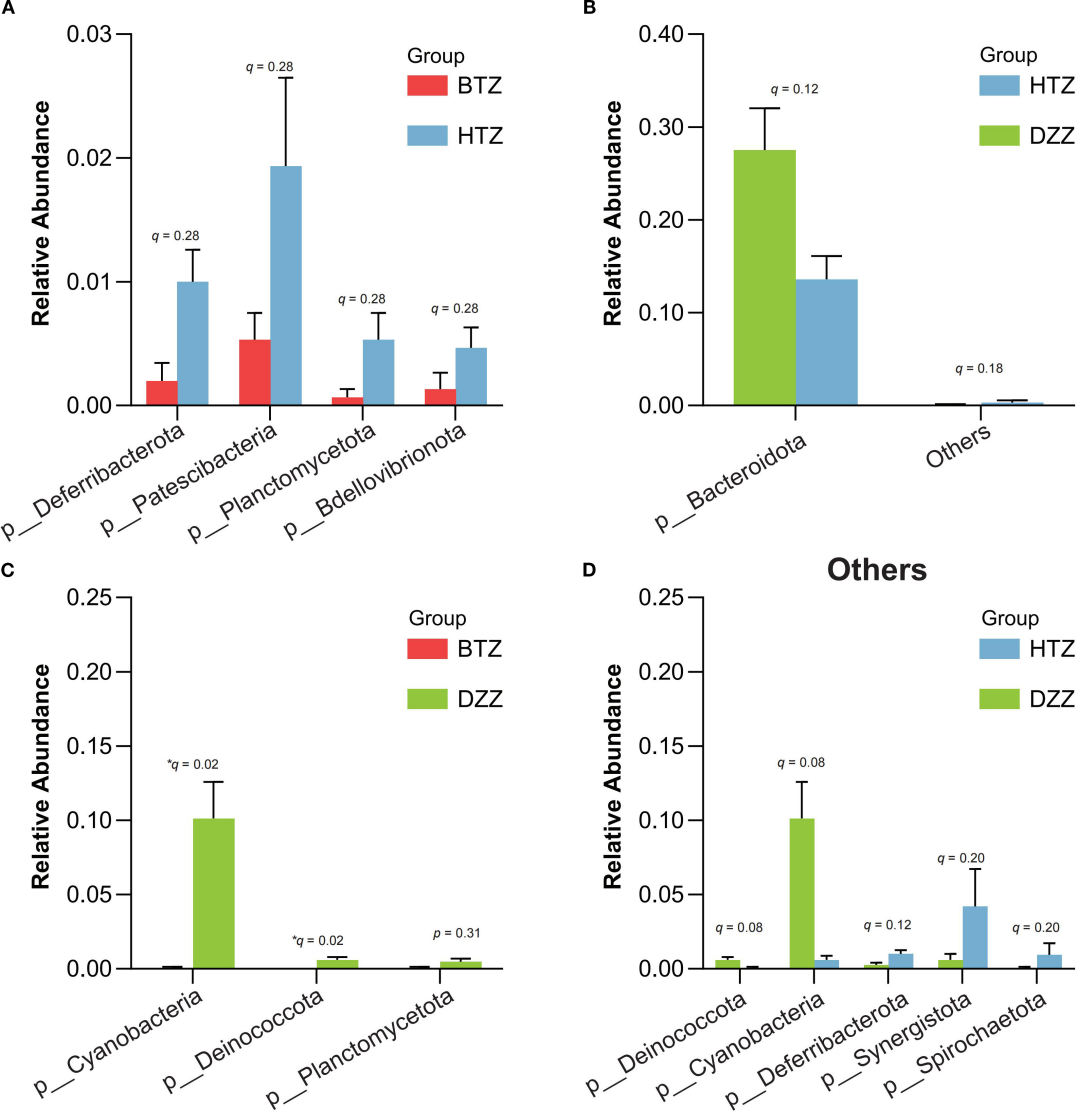
Comparison of microbial abundance at the phylum level. Error bars represent the standard error of the mean (SEM). Key findings are based on nominal p-values and should be interpreted with caution pending further validation: **(A)** HTZ had higher abundances of *Patescibacteria*, *Deferribacterota*, *Planctomycetota*, and *Bdellovibrionota* compared vs. BTZ. **(B, D)** Bacteroidota was lower in HTZ compared vs. DZZ. **(C)** DZZ had higher abundances of *Cyanobacteria* and *Deinococcota* compared vs. BTZ.

Sample clustering was performed using Bray-Curtis distances, and phylum-level stacked plots were drawn to better illustrate the differences and similarities among HTZ, BTZ, and DZZ. Additionally, cluster analysis revealed that the phylum-level stacked plots showed the dominant phyla in the intestinal microbiota of the three groups were *Firmicutes*, *Bacteroidota*, *Actinobacteriota*, and *Proteobacteria* (q < 0.05). The microbiota compositions of the HTZ and BTZ groups were more similar (**Supplementary Figure S1**). Through intergroup difference analysis, nominal differences (unadjusted p < 0.05) were observed for the phyla *Patescibacteria* (q = 0.28), Deferribacterota (q = 0.28), *Planctomycetota* (q = 0.28), and *Bdellovibrionota* (q = 0.28), which had higher relative abundances in the HTZ group compared to the BTZ group. However, these differences did not remain statistically significant after FDR correction (q > 0.05) and should be interpreted as potential trends warranting further investigation. Although the relative abundances of these phyla were low (< 2%), the biological relevance of these nominal findings remains uncertain. The phyla *Cyanobacteria* (q = 0.02), and *Deinococcota* (q = 0.02), exhibited statistically significant higher abundances in the DZZ group compared to the BTZ group. Furthermore, a nominal reduction (p = 0.019) in the abundance of *Bacteroidota* (q = 0.12) was observed in the HTZ group compared to the healthy control group, but this difference was not significant after multiple testing correction (**Figures 6**).

**Figure 7:**
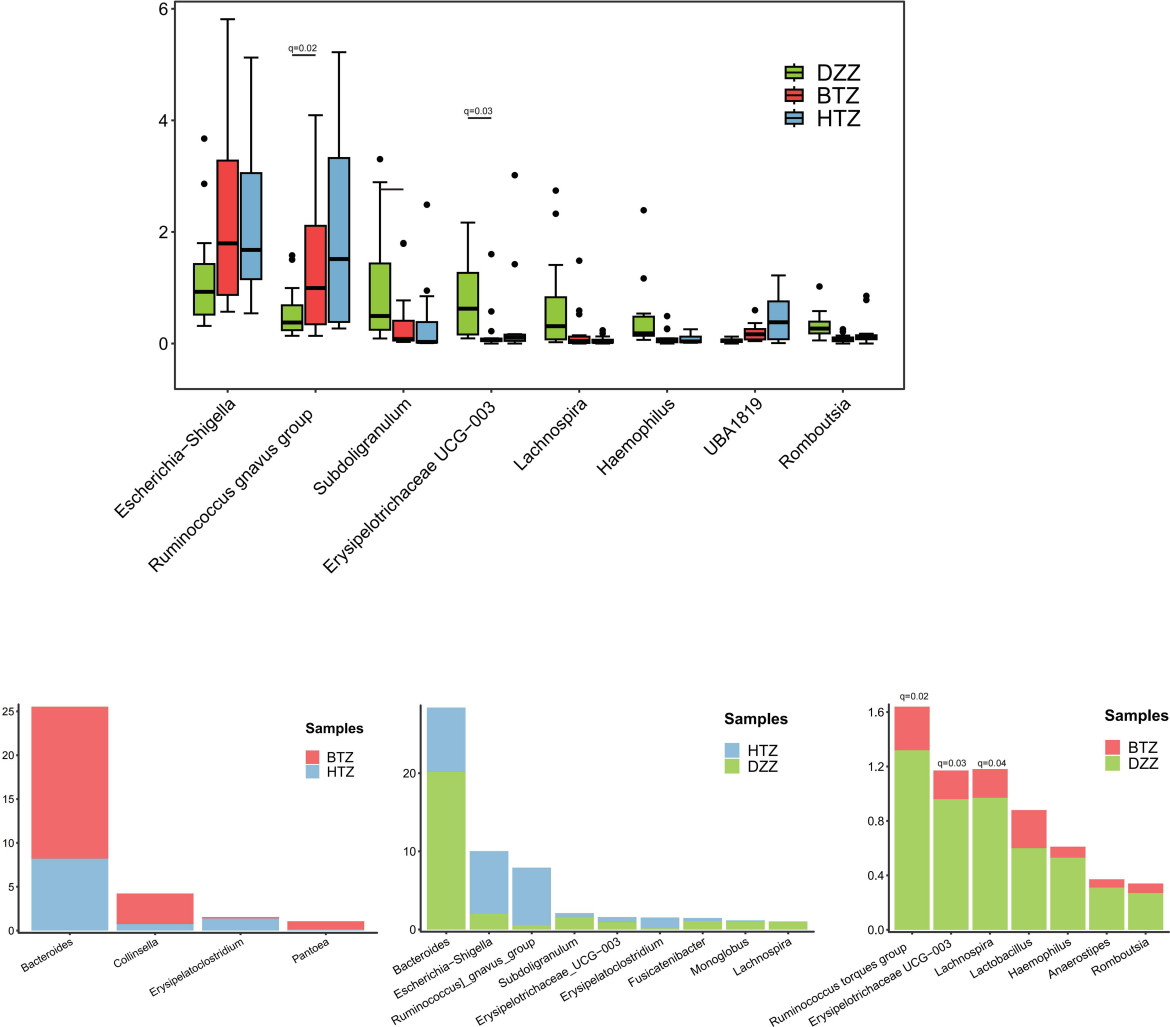
Comparison of microbial composition at the genus level. Bray-Curtis clustering revealed that HTZ and BTZ groups were more similar in genus-level microbial composition, clearly separated from the DZZ group. Compared to DZZ (controls), HTZ/BTZ showed significantly lower *Subdoligranulum, Lachnospira, Romboutsia, Haemophilus, Erysipelotrichaceae UCG-003* and nominally higher *UBA1819, Escherichia-Shigella, Ruminococcus gnavus group*, though only changes in *UBA1819* remained significant after FDR correction (q < 0.05). HTZ: nominally higher *Erysipelatoclostridium*/*Ruminococcus gnavus group* vs. DZZ; BTZ: significantly lower *Romboutsia/Erysipelotrichaceae UCG-003/Haemophilus* vs. DZZ. HTZ vs. BTZ: BTZ had nominally higher *Bacteroides*, *Collinsella*, *Pantoea*; HTZ had nominally higher *Erysipelatoclostridium*.

At the genus level, the Top 10 dominant genera in the intestinal microbiota of the HTZ, BTZ, and DZZ groups were: *Bacteroides*, *Bifidobacterium*, *Faecalibacterium*, *Escherichia-Shigella*, *Akkermansia*, *Ruminococcus gnavus group*, *Streptococcus*, *Megamonas*, *Klebsiella*, and *Ruminococcus*. Intergroup difference analysis using Kruskal-Wallis/Dunn’s test revealed that compared to DZZ, the relative abundances of genera such as *Subdoligranulum* (q = 0.03), *Lachnospira* (q = 0.01), *Romboutsia* (q = 0.01), *Haemophilus* (q = 0.01), and *Erysipelotrichaceae UCG-003* (q = 0.01) were significantly lower in HTZ and BTZ, while the relative abundances of genera such as *UBA1819* (q=0.01), *Escherichia-Shigella* (q = 0.08) and *Ruminococcus gnavus group* (q = 0.07) were nominally higher (unadjusted p < 0.05). Additionally, HTZ showed nominally higher abundances of genera *Erysipelatoclostridium* (q = 0.07) and *Ruminococcus gnavus group* (q = 0.12) compared to DZZ (unadjusted p < 0.05), while BTZ showed significantly lower abundances of genera *Romboutsia* (q = 0.02), *Erysipelotrichaceae UCG-003* (q = 0.03), and *Haemophilus* (q = 0.04) compared to DZZ. Between HTZ and BTZ, no comparisons survived FDR correction. However, nominal differences (unadjusted p < 0.05) were observed: BTZ had higher abundances of *Bacteroides* (q = 0.34), *Collinsella* (q = 0.36), and *Pantoea* (q = 0.51), while HTZ had higher abundances of *Erysipelatoclostridium* (q = 0.25). These trends suggest potential compositional distinctions that merit exploration in larger cohorts (**Figure 7**).

#### 3.3.3 LEfSe analysis

**Figure 8:**
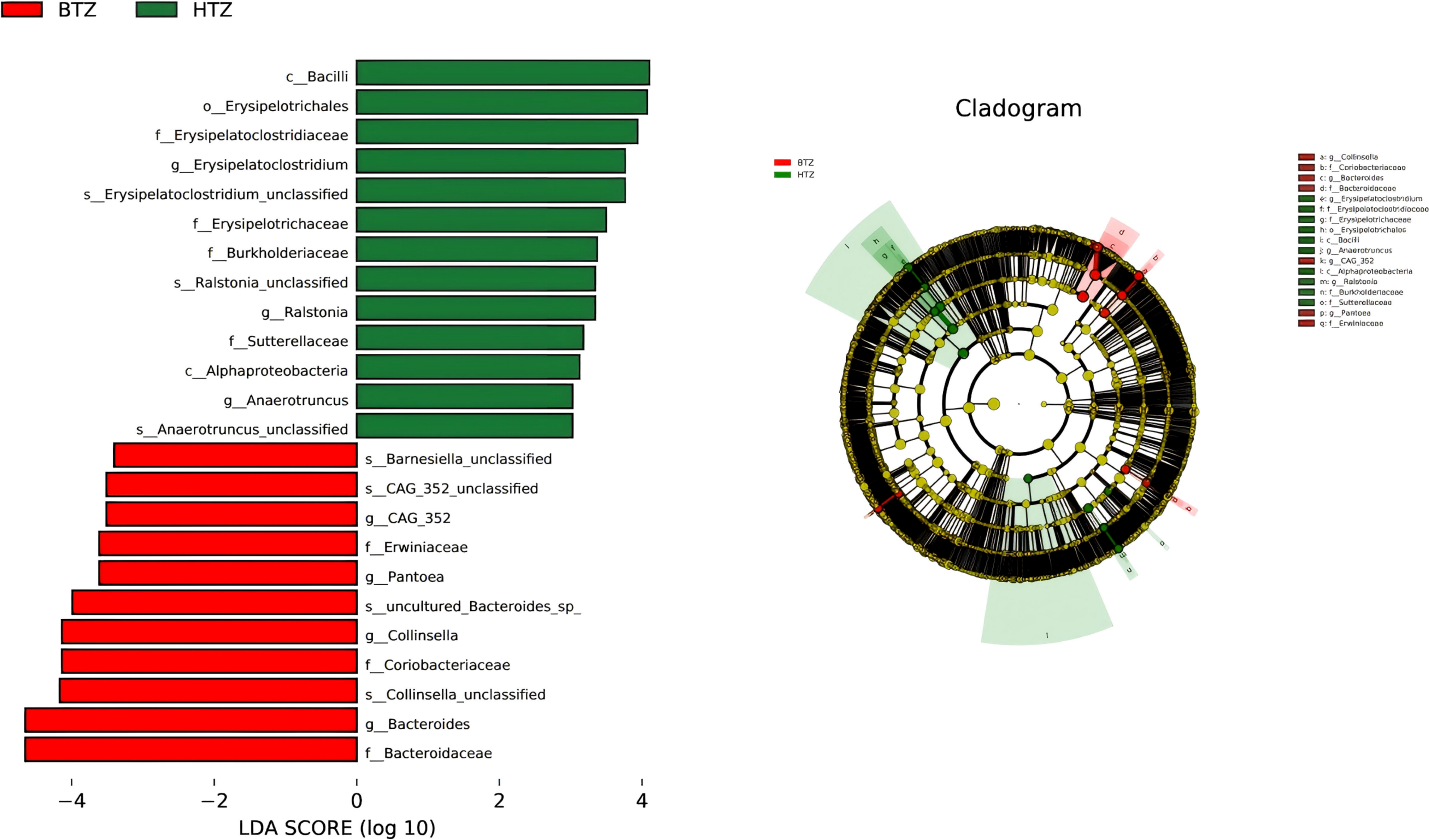
LEfSe analysis. LDA histogram (LDA > 3) and cladogram. Results are based on unadjusted p-values and represent potential biomarkers requiring further validation. HTZ-enriched taxa: *Bacilli*, *Erysipelotrichales*, *Erysipelatoclostridiaceae*, *Burkholderiaceae*, *Ralstonia*. BTZ-enriched taxa: *Bacteroidaceae*, *Collinsella*, *Pantoea*, *Erwiniaceae*. Cladogram shows taxonomic relationships of key discriminators.

The histogram of LDA values showed that 61 differential taxa (LDA score > 3, unadjusted p < 0.05) were observed among the HTZ, BTZ, and DZZ groups. However, after FDR correction, none of these taxa reached the threshold of statistical significance (q > 0.05). The LDA results thus represent unadjusted potential biomarkers that require validation in independent studies. Specifically, 15 differential taxa, including *Ruminococcus gnavus group*, *Bacilli*, *Erysipelotrichales*, and *Erysipelatoclostridiaceae*, had higher abundances in the HTZ group (unadjusted p < 0.05). Forty differential taxa, such as *Bacteroidota*, *Bacteroidia*, *Subdoligranulum*, *Eubacterium eligens group*, and *Lachnospira* unclassified, were more abundant in the intestinal microbiota of healthy individuals (unadjusted p < 0.05). Nine differential taxa, including *Escherichia-Shigella*, *Collinsella* unclassified, *Bacteroides uniformis*, and *Collinsella*, had higher abundances in the BTZ group (unadjusted p < 0.05). The Cladogram showed the significantly different taxa and their evolutionary branch relationships among the three groups. LEfSe analysis between HTZ and BTZ showed that 13 differential microbiota, including *Bacilli* (q = 0.31), *Erysipelotrichales* (q = 0.06), *Erysipelatoclostridiaceae* (q = 0.16), *Burkholderiaceae* (q = 0.16), and *Ralstonia* (q = 0.29), had nominally higher abundances (unadjusted p < 0.05) in the HTZ group, while 11 differential microbiota, such as *Bacteroidaceae* (q = 0.22), *Collinsella* (q = 0.36), *Pantoea* (q = 0.50), and *Erwiniaceae* (q = 0.35), had nominally higher abundances (unadjusted p < 0.05) in the BTZ group. The cladogram illustrates these potential taxonomic discriminators and their evolutionary relationships between HTZ and BTZ, though their statistical robustness is limited (**Figure 8**).

### 3.4 Predictive functional analysis

**Figure 9:**
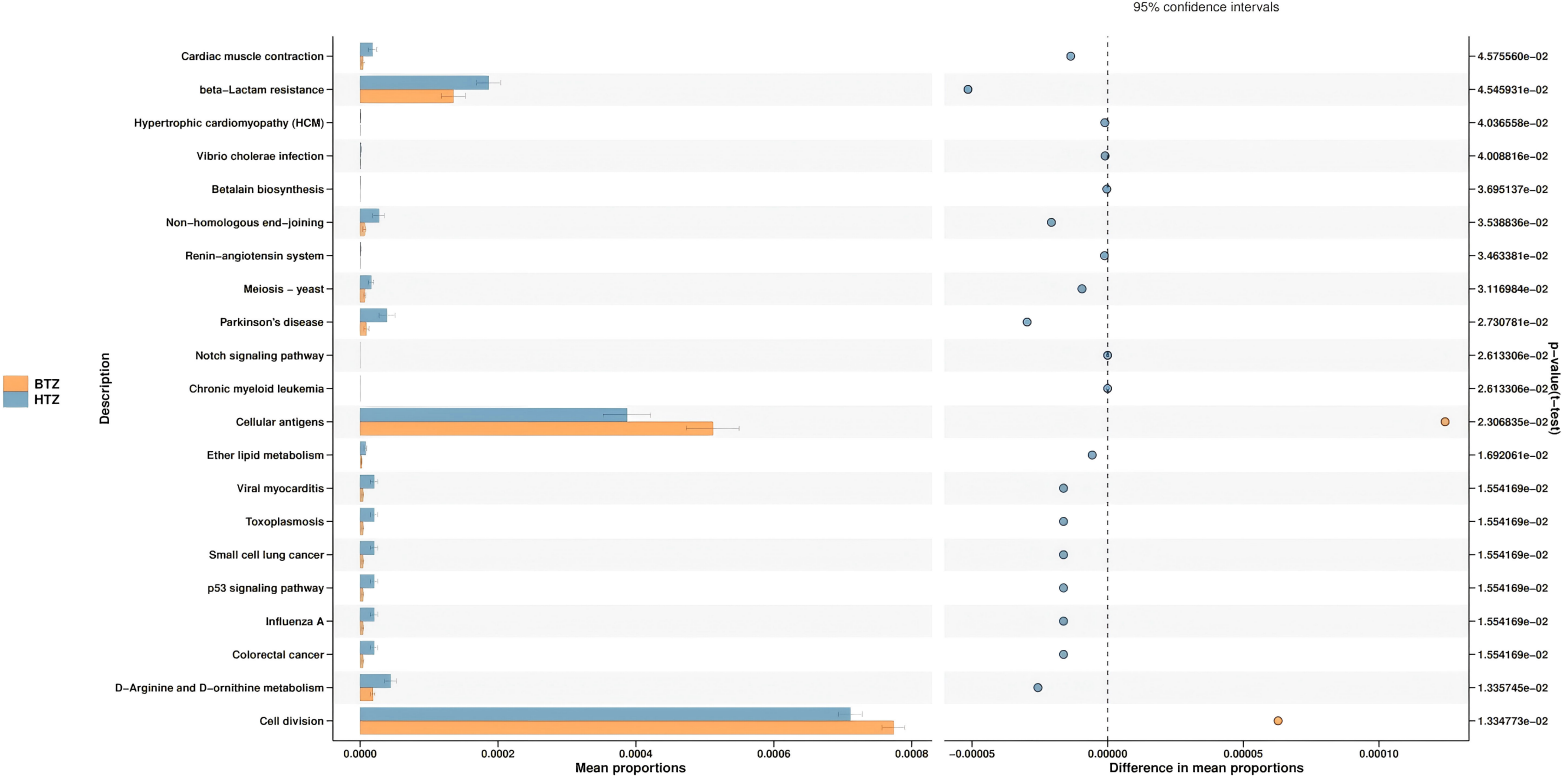
Predictive functional analysis. Key functional differences between HTZ and BTZ. HTZ showed enhanced pathways in cardiac muscle contraction, beta-lactam resistance, and Parkinson’s disease-related metabolism. Reduced functions in HTZ included cellular antigen processes and cell division.

The species’ functions in the gut microbiota of both the groups were predicted and analyzed based on the amplified sequencing data. Using t-test for differential analysis with Benjamini-Hochberg correction for multiple testing, PICRUSt2-based functional prediction of the intestinal microbiota in MHD patients showed that compared to BTZ, HTZ had significantly enhanced microbial functions in cardiac muscle contraction (q = 0.05), beta-lactam resistance (q = 0.05), non-homologous end-joining (q = 0.04), Parkinson’s disease (q = 0.03), and D-arginine and D-ornithine metabolism (q = 0.01), while significantly decreased functions in cellular antigens (q = 0.02) and cell division (q = 0.01) (**Figure 9**).

## 4 Discussion

In this study, we found that tongue coating thickness in hemodialysis patients is associated with distinct differences in gut microbiota composition, diversity, and predicted function. Both thick tongue coating (HTZ) and thin tongue coating (BTZ) groups exhibited gut dysbiosis compared to healthy controls (DZZ), but they also differed from each other in specific microbial features. While several taxonomic comparisons between patient groups (HTZ vs. BTZ) did not retain statistical significance after rigorous multiple testing correction, the persistence of nominal differences (unadjusted p < 0.05) and consistent trends across analyses suggest potential, albeit subtle, distinctions in their gut microbial ecosystems that align with the tongue coating phenotype.

A Venn diagram of ASVs illustrated that while all three groups shared a core of 667 ASVs, each patient group had unique taxa, with HTZ showing the highest total ASV count (2970) compared to BTZ (2487) and DZZ (2964). This indicates that HTZ patients harbored a broader range of microbial species (including possibly opportunistic taxa) relative to BTZ. Correspondingly, α-diversity measures (Observed species, Shannon, Simpson indices) were significantly lower in BTZ than in healthy controls, whereas HTZ showed intermediate diversity. DZZ exhibited greater species richness and evenness than BTZ (q < 0.05), while no significant difference was noted between HTZ and BTZ in these indices. These results suggest that BTZ patients have a more depleted gut microbiome diversity compared to healthy individuals, whereas HTZ patients, despite severe illness, did not experience the same loss of diversity. However, β-diversity analysis revealed that both HTZ and BTZ groups cluster closely together and separate from DZZ (**Figure 4**). Although α-diversity indices showed no statistically significant difference between HTZ and DZZ, this likely reflects preserved overall richness and evenness in some HD patients with thick coatings. However, our β-diversity and taxonomic analyses revealed clear compositional shifts between these two groups, including the nominal enrichment of pathogenic genera in HTZ. This suggests that tongue coating phenotype in HD patients corresponds more strongly with microbiota structure than with global diversity metrics. The PCoA plots showed only partial overlap between patient and control samples, with HTZ and BTZ microbiotas more similar to each other than either is to healthy controls. This indicates that both patient groups share a common dysbiotic footprint characteristic of maintenance hemodialysis, distinct from the normal gut microbiome, even though HTZ and BTZ differ in certain nuances.

When comparing gut microbiota composition, we observed broad shifts at multiple taxonomic levels associated with tongue coating thickness. At the phylum level, the gut communities of all groups were dominated by *Firmicutes*, *Bacteroidota*, *Actinobacteriota*, and *Proteobacteria*, consistent with a typical human gut profile. Nonetheless, several lower-abundance phyla showed significant differences between groups (**Figure 6**). At the phylum level, the nominal increases in Patescibacteria, Deferribacterota, Planctomycetota, and Bdellovibrionota in HTZ versus BTZ, though not statistically robust after correction, are intriguing. These phyla are often minor constituents or considered to be associated with specific ecological niches or dysbiotic states. Their potential elevation in HTZ could hint at a more disrupted or specialized microbial community deserving of further scrutiny. In contrast, healthy controls showed statistically significant higher abundances (q < 0.05) of *Cyanobacteria* (likely reflecting dietary plant chloroplasts or commensal Cyanobacterial lineages) and *Deinococcota* than BTZ. The BTZ group’s near absence of these taxa may be due to dietary differences or a generally reduced microbial complexity in patients. Additionally, the major phylum *Bacteroidota* was nominally depleted in HTZ compared to healthy controls. This aligns with the genus-level finding of reduced Bacteroides and points toward a potential loss of important commensal, fiber-degrading bacteria in thick-coated patients. Taken together, these phylum-level changes suggest that HTZ microbiomes are skewed toward uncommon or potentially pathogenic phyla, whereas BTZ microbiomes show loss of some normally occurring phyla found in healthy guts.

At the genus level, dysbiosis patterns were evident in both patient groups relative to controls, as well as between HTZ and BTZ themselves. The overall top ten genera in all groups included *Bacteroides*, *Bifidobacterium*, *Faecalibacterium*, *Escherichia-Shigella*, *Akkermansia*, *Ruminococcus gnavus group*, *Streptococcus*, *Megamonas*, *Klebsiella*, and *Ruminococcus* (torques group), indicating that many core gut genera are present across the spectrum of health and disease. However, many beneficial genera were significantly under-represented in both HTZ and BTZ patients compared to healthy DZZ, consistent with the known impact of uremia on the gut microbiome (Simões-Silva et al., 2018). Specifically, both patient groups showed significant reductions in SCFA-producing and fiber-fermenting bacteria such as *Subdoligranulum, Lachnospira, Romboutsia, Haemophilus and Erysipelotrichaceae UCG-003* compared to controls (q < 0.05). Concurrently, opportunistic or proteolytic genera were elevated: for example, *UBA1819*, *Escherichia-Shigella* and the *Ruminococcus gnavus group* were nominally higher in both HTZ and BTZ vs. DZZ, though only changes in *UBA1819* remained significant after FDR correction (q < 0.05). These shifts mirror hallmark features of CKD-related gut dysbiosis - a loss of commensals that produce beneficial metabolites (like butyrate) and an overgrowth of bacteria that produce endotoxins and uremic toxins (Beau et al., 2025). Indeed, *Escherichia-Shigella* (an *Enterobacteriaceae* member) can generate endotoxin and indoxyl sulfate precursors (Hayashi et al., 2018; Yu et al., 2025), while *R. gnavus* is known for mucin degradation and pro-inflammatory polysaccharide production (Crost et al., 2016); their increase in both HTZ and BTZ underscores a gut environment inclined toward inflammation and toxin production in MHD patients. Notably, some differences with healthy controls were unique to each patient subgroup. HTZ patients showed a nominal increase in *Erysipelatoclostridium (genera in the Erysipelotrichaceae family)* and *Ruminococcus gnavus group* compared to DZZ, suggesting that certain pro-inflammatory or putrefactive taxa may preferentially expand when the tongue coating is thick. Meanwhile, BTZ patients had a greater loss of *Romboutsia*, *Erysipelotrichaceae UCG-003*, and *Haemophilus* compared to DZZ; *Romboutsia* is a beneficial anaerobe that can utilize lactate and, through cross-feeding interactions with other microbes, promote butyrate production (Chen et al., 2025)- indicating that even thin-coated patients suffer a deficit of key commensals relative to healthy individuals. Thus, a thin tongue coating should not be equated with a normal microbiome; rather, it represents a milder dysbiosis where some harmful changes are present but perhaps to a lesser degree than in thick-coated patients.

Direct comparison between HTZ and BTZ groups revealed a subset of genera that differentiate the two dysbiotic states (**Figure 7**). At the genus level, the pattern of dysbiosis was more clearly evident when comparing both patient groups to healthy controls, with several changes, such as the increase in *Escherichia-Shigella* and decrease in *Erysipelotrichaceae UCG-003*, surviving multiple testing correction. The direct comparison between HTZ and BTZ yielded nominal differences that, while requiring cautious interpretation, paint a biologically plausible picture. For instance, the trend of higher *Bacteroides* in BTZ and higher *Erysipelatoclostridium* in HTZ is consistent with the notion that a thin coating might be associated with a relatively better-preserved capacity for fiber fermentation, while a thick coating might indicate a shift towards pro-inflammatory or putrefactive taxa, but these specific HTZ vs BTZ contrasts were nominal and require validation in larger cohorts. The LEfSe analysis, though comprised of unadjusted p-values, further supports this narrative by identifying coherent groups of taxa (e.g., *Erysipelotrichales* and *Burkholderiaceae* in HTZ; *Bacteroidaceae* and *Erwiniacea*e in BTZ) that differentiate the two groups (**Figure 8**). The convergence of these trends from different analytical approaches strengthens the hypothesis that tongue coating reflects meaningful, albeit subtle, variations in the gut microbiome of MHD patients.

The functional potential of the gut microbiota, inferred via PICRUSt2 analysis, also differed between HTZ and BTZ, though in subtler ways (**Figure 9**). However, these functional predictions are derived from 16S rRNA gene data and are inherently speculative. They lack the resolution of direct functional profiling methods (e.g., metagenomics or metabolomics) and should be interpreted with caution, particularly in disease-altered microbiomes such as ESRD where taxonomic-functional relationships may be disrupted. Notably, HTZ showed higher representation of gene pathways related to cardiovascular diseases and infections compared to BTZ. For instance, pathways such as “Cardiac muscle contraction”, “Hypertrophic cardiomyopathy”, and infectious disease pathways (e.g., “Vibrio cholerae infection”) were enriched in the HTZ microbiome, whereas these were less prominent in BTZ. HTZ also had higher predicted abundances of pathways involved in various metabolic diseases (“disease metabolism”). These enriched functions suggest that the HTZ-associated microbiota may contribute to a more pro-inflammatory or pathological metabolic milieu – for example, harboring more genes related to toxin production or host-interactive pathways that could influence cardiac and immune function. In contrast, certain fundamental cellular processes were relatively under-represented in HTZ versus BTZ. Our data indicate that pathways involved in basic cellular functions (for example, DNA repair mechanisms like non-homologous end joining, and cell division-related pathways) were predicted to be reduced in HTZ. One interpretation is that the microbial community in HTZ might be skewed away from maintaining normal cellular homeostasis and towards stress or virulence-associated metabolism, perhaps reflecting a community under or causing host stress. It is important to emphasize that these functional predictions are hypothetical – they provide hints that HTZ microbiomes could be functionally more disruptive (e.g., contributing to cardiovascular risk or infections), whereas BTZ microbiomes might retain relatively more “basic” functionality – but they need experimental validation.

The observed differences in gut microbiota between thick and thin tongue coating patients beg the question of mechanism and causality. One plausible explanation involves the oral-gut microbial axis. Tongue coating is essentially an oral microbial biofilm; patients with thick coatings likely harbor an overgrowth of oral microbes (including anaerobes and possibly yeast or others) on the tongue surface (Guo et al., 2025). These microbes, or their metabolites, can be continuously swallowed and thus influence the gut microbiota composition downstream. Indeed, our team’s previous work showed significant differences in the oral microbiota between HTZ and BTZ patients. For example, we found higher abundance of certain oral genera (like *Prevotella* and *Megasphaera*) in thick-coated patients. It has been reported that the oral and gut microbiomes can mirror each other in specific ways – microbes prevalent in the tongue coating often appear in the gut, and changes in oral microbiota can accompany changes in gut microbiota in various diseases (Deng et al., 2024). Previous studies have documented correlations such as the relative abundance of *Prevotella* being positively linked between tongue coating and feces in healthy individuals (Guo et al., 2022). In disease states (e.g., diarrhea-predominant IBSor MAFLD), shifts in tongue-coating microbiota parallel alterations in the gut community (Lu et al., 2022; Tang et al., 2023). Moreover, systemic illnesses (like COVID-19 or schizophrenia) are known to perturb both oral and gut microbiomes simultaneously, and translocation of oral bacteria to the gut tends to increase with age or immune compromise (Murray et al., 2021; Jin et al., 2022; Ghorbani, 2023; Zhang et al., 2023). Given this context, it is reasonable to speculate that a thick tongue coating could be contributing to gut dysbiosis by serving as a reservoir for microbes that colonize or influence the intestinal environment. For instance, *Erysipelatoclostridium* and other *Erysipelotrichaceae* found enriched in HTZ gut are also known inhabitants of the oral cavity; an overgrowth on the tongue could seed the gut continually. Likewise, the presence of *Ralstonia* or *Pantoea* might originate from environmental exposure in the oral cavity (water, food) that, under normal conditions, would not persist in the gut but in an altered host environment they manage to survive. Additionally, from a Traditional Chinese Medicine (TCM) perspective, a thick tongue coating is thought to indicate internal “dampness” or stagnation in the gastrointestinal system (Guo et al., 2025). Biomedically, this concept may translate to slower gut motility, altered pH, or retention of food residues - conditions that can foster microbial overgrowth. It’s conceivable that HTZ patients have underlying GI functional differences (such as more frequent constipation or delayed transit) which could allow certain bacteria to proliferate excessively, whereas BTZ patients might have relatively faster transit or less substrate accumulation limiting microbial expansion. We did not specifically measure motility or oral-gut transmission in this study, so these mechanisms remain speculative. Nevertheless, the oral-gut axis hypothesis is supported by our finding that many of the taxa distinguishing HTZ and BTZ are not independent random occurrences but belong to known oral-related or dysbiosis-associated groups. Future studies integrating oral microbiome sequencing and gut transit measurements could elucidate this causal chain more clearly.

From a clinical standpoint, our findings carry potential practical significance. Tongue coating is a simple, noninvasive clinical observation that could serve as a surrogate marker for gut microbiota status in hemodialysis patients. If a thick tongue coating correlates with a greater degree of gut dysbiosis - characterized by higher loads of pro-inflammatory bacteria and altered metabolic potential - it might alert clinicians to patients who are at risk of GI complications or systemic inflammation stemming from the gut. Conversely, a thin tongue coating, while not indicating a normal microbiota, might correspond to a relatively milder dysbiosis. This concept aligns with the tenets of TCM, but here we provide scientific evidence that tongue appearance does reflect internal micro-ecology. Tongue coating thickness could thus be used as a screening tool or adjunct in evaluating patient health, supporting the notion of it as a “window” into the gut microbiome (Zhu et al., 2025). For example, an MHD patient presenting with a notably thick, greasy tongue coating might benefit from early interventions targeting the gut flora - such as prebiotic fiber supplementation, probiotics, or diet modifications (e.g. increasing fermentable fiber or reducing protein load to curb proteolytic bacteria). In contrast, a patient with a thin coating might be monitored for loss of beneficial microbes and could benefit from strategies to increase microbial diversity (such as a more plant-rich diet or specific commensal probiotics).

## 5 Limitations and future directions

While this study provides novel insights into the microbiota-tongue coating relationship, several limitations warrant consideration. First, the cross-sectional design precludes causal inference, necessitating longitudinal studies to track microbiota dynamics and clinical outcomes. Second, the functional predictions are based on computational modeling rather than direct experimental validation, requiring follow-up *in vitro*/*in vivo* studies to mechanistically validate pathway alterations. Third, the sample size, while sufficient to detect larger effects against healthy controls, may have limited the power to detect more subtle but biologically relevant differences between the HTZ and BTZ groups after stringent multiple testing correction. The consistent trends observed (unadjusted p < 0.05) despite the lack of FDR significance highlight the need for larger, specifically powered studies to confirm or refute these potential associations. Such inferences are limited by the incomplete representation of gene functions in reference databases and may not accurately reflect the true metabolic potential of complex or dysbiotic microbiomes, such as those in ESRD patients. Caution is warranted when interpreting KEGG pathway enrichments as direct indicators of disease mechanisms. Lastly, the generalizability of findings may be limited by the specific HD patient population studied, emphasizing the need for multi-center cohorts with diverse ethnic and dietary backgrounds. Fourth, we did not further stratify healthy controls (DZZ) by tongue coating thickness, as none of them exhibited a thick coating (TCT ≥ 24) or a thin coating (TCT ≤ 13). All healthy individuals had moderate TCT scores (14–23), representing physiologically normal variation. This limited stratification helps avoid overinterpretation but may restrict the exploration of subtle oral–gut microbiome associations in non-HD populations. Future studies with larger healthy cohorts are warranted to determine whether similar associations hold in the general population. Future research should prioritize integrative approaches combining metagenomics, metabolomics, and proteomics to unravel the complex interactions between microbiota, host metabolism, and tongue coating physiology. Additionally, interventional trials evaluating probiotics, prebiotics, or fecal microbiota transplantation (FMT) in HTZ patients could establish whether microbial reconstitution alleviates clinical symptoms and improves long-term outcomes in HD.

## 6 Conclusion

This study reveals that tongue coating thickness in maintenance hemodialysis patients reflects distinct subtypes of gut microbiota dysbiosis. Thick coatings are associated with pro-inflammatory microbial features, while thin coatings indicate lower diversity yet partial preservation of commensals. These patterns suggest that tongue appearance may serve as a visible indicator of internal microbial states, offering clinically relevant insights into systemic health. Integrating traditional tongue diagnosis with microbiome profiling could support personalized strategies to improve gastrointestinal and metabolic outcomes in dialysis care.

## Data Availability

The datasets presented in this study can be found in online repositories. The names of the repository/repositories and accession number(s) can be found below: https://www.ncbi.nlm.nih.gov/, PRJNA1267578.
