# Supplementary figures and images for: Thick vs. thin tongue coatings in hemodialysis patients: unveiling gut microbiome dysregulation and systemic health implications
Source: Front Cell Infect Microbiol. 2025 Sep 29;15:1640429. doi: 10.3389/fcimb.2025.1640429 (PMC12515952; doi:10.3389/fcimb.2025.1640429)

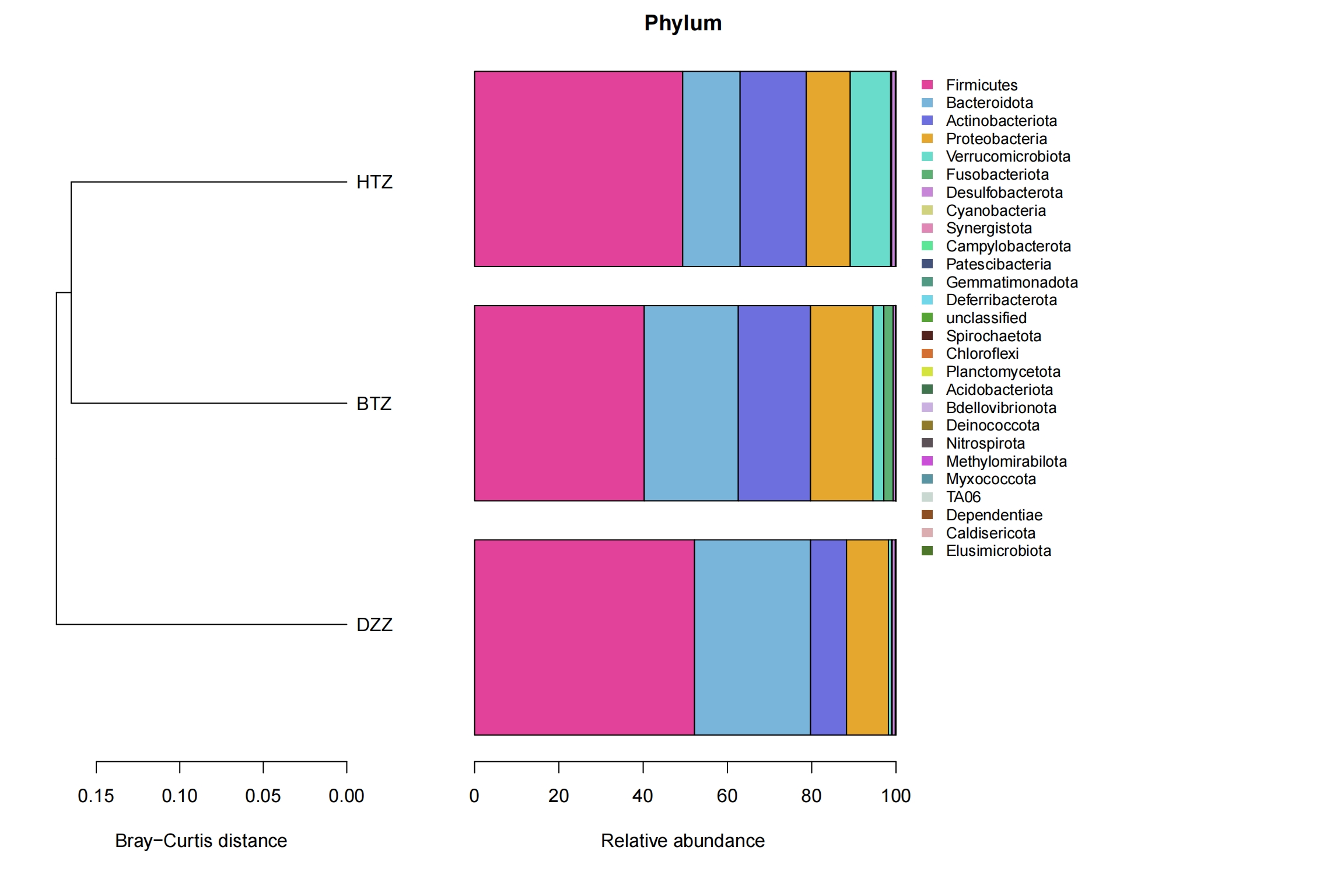

Supplement: Supplementary file 1 [file Image1.jpeg]
